# Left ventricular strain measured by feature-tracking cardiac magnetic resonance imaging and 2D speckle-tracking echocardiography in chronic ischemic heart disease: an intermodality agreement study

**DOI:** 10.1186/s44348-026-00065-w

**Published:** 2026-04-09

**Authors:** Shimaa Sayed Khidr, Ahmed Abdel-Galeel, Mohamed Abdellatif, Abdulrahman Hamdan, Yehia Taha Kishk

**Affiliations:** 1 Department of Cardiovascular Medicine, Faculty of Medicine, Assiut University, Assiut, Egypt; 2 Department of Cardiovascular Medicine, Faculty of Medicine, Luxor University, Luxor, Egypt; 3 Aswan Heart Centre, Magdi Yacoub Foundation, Aswan, Egypt

**Keywords:** Myocardial strain, Feature-tracking cardiac magnetic resonance, Speckle-tracking echocardiography, Ischemic cardiomyopathy, Ventricular function

## Abstract

**Background:**

Global longitudinal strain (GLS) is a valuable tool for assessing left ventricular (LV) systolic function, detecting subclinical dysfunction earlier than classic ejection fraction. Two-dimensional speckle-tracking echocardiography (2D-STE) is widely used due to its accessibility and high temporal resolution, whereas feature-tracking cardiac magnetic resonance (FT-CMR) offers superior spatial resolution and reproducibility. In this study, we assess the relationships between longitudinal strain measurements obtained by 2D-STE and FT-CMR in patients with chronic ischemic heart disease (IHD).

**Methods:**

Fifty-five patients with IHD and left ventricular ejection fraction (LVEF) ≤ 49% underwent 2D-STE and FT-CMR at least 3 months after an acute coronary event. Global and segmental longitudinal strain for all 17 myocardial segments was measured using both modalities. Pearson correlation and Bland–Altman analysis were used to assess correlation and agreement, respectively.

**Results:**

GLS showed a strong correlation between 2D-STE and FT-CMR (r = 0.793, P < 0.001), with a mean difference of 0.98% and limits of agreement from –3.2% to + 5.1%. Segmental strain demonstrated greater variability (r = 0.03 to 0.47), with the best agreement in mid and apical segments and greatest discrepancies at the basal level.

**Conclusions:**

In patients with IHD and reduced LVEF, GLS obtained by FT-CMR and 2D-STE showed strong correlation and acceptable overall agreement. However, the relatively wide limits of agreement and variability in segmental strain, particularly in basal regions, indicate that the two methods are not fully interchangeable for individual assessment and follow-up. Both techniques reflect similar physiological trends but differ in absolute values, requiring caution in regional strain interpretation.

**Supplementary Information:**

The online version contains supplementary material available at 10.1186/s44348-026-00065-w.

## Background

Left ventricular (LV) systolic function is a critical determinant of cardiovascular health, traditionally assessed by LV ejection fraction (LVEF). However, LVEF has limitations, including load dependency and insensitivity to early myocardial dysfunction. Global longitudinal strain (GLS) is a sensitive and reproducible marker that can identify early myocardial impairment, even with normal values of LVEF [1].

Two-dimensional speckle-tracking echocardiography (2D-STE) is an accessible and cost-effective modality for GLS assessment. It is well suited for routine clinical use and offers high temporal resolution. However, its accuracy can be affected by several factors, including image quality, echo window, and operator experience [2].​

Feature-tracking cardiac magnetic resonance (FT-CMR) utilizes routine CMR cine images to measure myocardial deformation. It provides higher spatial resolution than 2D-STE and is not affected by acoustic windows. Recent studies demonstrated its reproducibility and potential for comprehensive myocardial assessment in clinical practice. [3]. However, it is more expensive and less accessible than 2D-STE.

Although both modalities offer distinct advantages and limitations, discrepancies have been reported between 2D-STE and FT-CMR measurements of GLS, particularly at the segmental level. Evaluating the degree of agreement and correlation between these techniques is crucial before considering their interchangeable use in clinical practice, especially in patients with ischemic heart disease (IHD) and reduced LVEF. This patient group exhibits complex myocardial characteristics, including varying degrees of fibrosis, scarring, and remodeling. Accurate assessment of both global and regional myocardial function is therefore essential for evaluating viability and identifying scar location. However, studies directly assessing intermodality agreement in this specific population remain limited [2].​

In this study, we evaluate the relationships between GLS and segmental longitudinal strain values derived from FT-CMR and 2D-STE in a specific patient group with IHD with impaired LVEF. We provide insights into their comparative utility in this important clinical scenario, in which strain studies are being utilized more frequently to guide clinical decisions.

## Methods

### Ethics statement

This study was approved by the Institutional Review Board of Faculty of Medicine, Assiut University (No. 17200538). Written informed consent was obtained from all participants before inclusion in the study. All procedures conformed to the ethical standards of the institutional and national research committee and with the 1964 Declaration of Helsinki and its later amendments.

### Study design and patients

This cross-sectional observational study included 55 consecutively recruited patients with IHD, confirmed by coronary angiography, who presented to Assiut University Heart Hospital for viability assessment between July 2022 and November 2023. Patients were eligible for inclusion if they underwent both CMR and echocardiography within one week of each other, both performed at least 3 months after their most recent acute event, and up to 12 months, during which time they remained clinically stable. Patients with nonischemic cardiomyopathy, poor echocardiographic window, or contraindications to CMR were excluded.

Demographic criteria, risk factors, and baseline clinical and angiographic data were recorded.

Coronary angiography was performed and evaluated by experienced interventional cardiologists at our institution, with significant coronary artery disease defined as visual thresholds of diameter reduction, ≥ 50% diameter stenosis in the left main coronary artery, and ≥ 70% diameter stenosis in major epicardial coronary arteries (e.g., left anterior descending, left circumflex, and right coronary artery) [4].

All patients underwent detailed echocardiographic assessment and CMR study, including images obtained after gadolinium-based dye injection, both of which were performed within 1 week.

### Imaging protocols and analysis

#### 2D-STE for longitudinal strain analysis

Echocardiographic examination was performed by an experienced echocardiographer who was blinded to the clinical and other imaging results using a GE-Vingmed Vivid-S5 ultrasound machine (GE Medical System), with a 2.5–3.5 MHz transducer. Myocardial segments meeting predefined image quality criteria, classified as “good” or “acceptable,” were included in the analysis. Left ventricular GLS was assessed using 2D-STE, performed from the standard apical four-, two-, and three-chamber long-axis views. Images were electrocardiography-gated and acquired with a frame rate of 50 to 90 frames per second over three cardiac cycles and analyzed offline using Echopac ver. 112 (GE Healthcare). For each apical view, the endocardial border was manually traced at end-systole, and the region of interest was adjusted to include the full myocardial thickness. The software automatically tracked myocardial motion throughout the cardiac cycle, generating longitudinal strain curves for each segment. Tracking quality was visually assessed and manually corrected when necessary. GLS was calculated as the average of the peak systolic longitudinal strain values from all 17 myocardial segments, derived from the three apical views. GLS is expressed as a negative percentage, reflecting myocardial shortening in the longitudinal direction during systole.

**Fig. 1:**
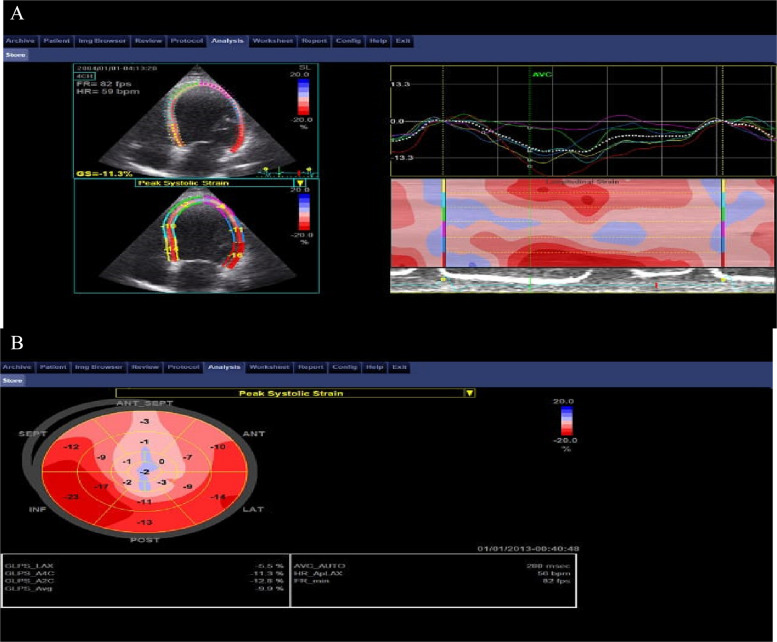
Curves (**A**) and Bull’s eye (**B**) displays of the segmental longitudinal strain measured by 2D speckle-tracking echocardiography in apical views

Segmental strain was assessed by examining the individual peak longitudinal strain values for each of the 17 segments based on the standard American Heart Association (AHA) model, as shown in Fig. 1 [5].

Intraobserver and interobserver variability were assessed in about 15% of randomly selected cases using intraclass correlation coefficients (ICCs) and Bland–Altman analysis.

### LGE-CMR imaging and FT-CMR for longitudinal strain analysis

The CMR studies were performed using a 1.5-T magnetic resonance imaging scanner (Philips Ingenia Release 4.1.3.0, Philips Medical Systems) at Assiut University Heart Hospital, using a phased array cardiac receiver coil. Standard cine steady-state free precession images were acquired in the two-, three-, and four-chamber long-axis views. A stack of short-axis images for volumetric and functional assessment was taken (Repetition time/Echo time, 3.1 ms/1.5 ms; flip angle, 70°; Field of view, 300 mm; voxel size, 1.97/2.05/8.00 mm; 8-mm slice thickness with no gaps for short-axis images). Late gadolinium enhancement (LGE) imaging was acquired with phase-sensitive inversion recovery in two-, three-, and four-chamber views, together with three to five short-axis slices. Viable segments were defined as those with < 50% subendocardial enhancement [6].

FT-CMR analysis was conducted using cine long-axis views and processed offline with Segment ver. 4.0 (Medviso).

**Fig. 2:**
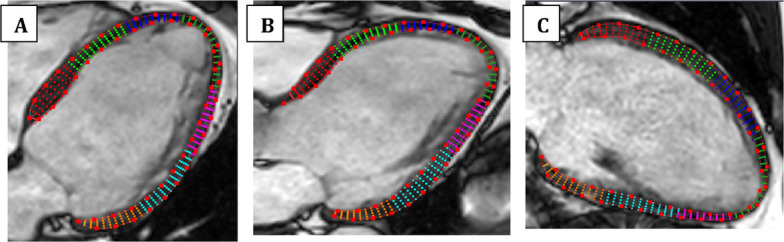
Feature-tracking cardiac magnetic resonance analysis in (**A**) four-chamber view, (**B**) three-chamber view, and (**C**) two-chamber view

**Fig. 3:**
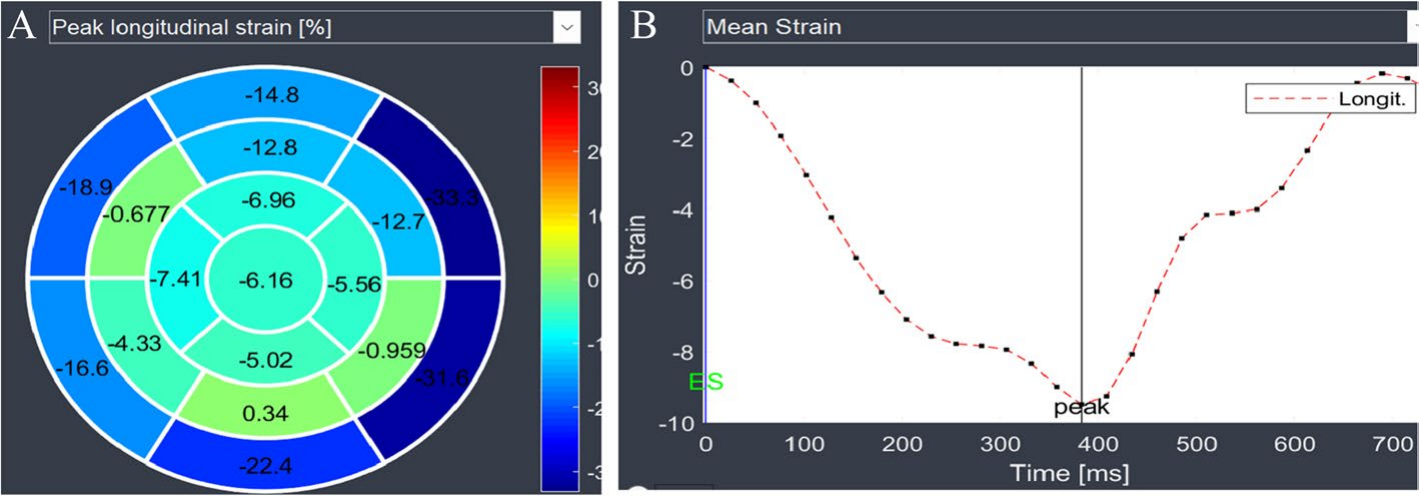
Bull’s eye displays (**A**) and curves (**B**) of the segmental longitudinal strain measured by feature-tracking cardiac magnetic resonance show much more reduced values in the nonviable segments

The endocardial and epicardial borders were manually delineated at end-diastole. The software then applied a nonrigid, elastic image registration algorithm to automatically track myocardial motion throughout the cardiac cycle, generating strain curves for each of the 17 segments according to the AHA model. Automated contour propagation was reviewed and corrected when necessary. Peak systolic longitudinal strain values were recorded for each segment, and GLS was calculated as the average of these values, as shown in Figs. 2 and 3 [7].

### Statistical analysis

Continuous variables were expressed as mean ± standard deviation (SD). Paired comparisons between strain values derived from 2D-STE and FT-CMR were conducted using the paired Student t-test. Agreement between modalities was assessed using Bland–Altman analysis, which included calculation of the mean bias and 95% limits of agreement (LoAs) [8]. The correlations of 2D-STE–derived longitudinal strain values with FT-CMR–derived longitudinal strain were performed with Pearson correlation test. Correlation strength was interpreted as follows: poor (< 0.50), moderate (0.50–0.75), good (0.75–0.90), and excellent (> 0.90) [9]. A P-value of < 0.05 was considered statistically significant. Intraobserver and interobserver variability were assessed using the intraclass correlation coefficient and Bland–Altman analysis, with an ICC value of > 0.75 considered to indicate good and > 0.90 excellent reproducibility. All statistical analyses were performed with IBM SPSS ver. 19.0 (IBM Corp).

## Results

### Baseline characteristics

This study included 55 patients with a mean age of 58.2 ± 9.6 years, of whom 85% were male. Baseline characteristics, basic echocardiographic, CMR, and angiographic data are presented in Table 1.

**Table 1:** Baseline clinical, angiographic, and imaging characteristics (n = 55)

Baseline characteristic	Value
Age (yr)	58.2 ± 9.6
Sex	
Male	47 (85.5)
Female	8 (14.5)
Comorbidity	
Diabetes mellitus	24 (43.6)
Hypertension	19 (34.5)
Body surface area (m^2^)	1.886 ± 0.148
Angiographic data	
Left main disease	5 (9.1)
Left anterior descending (diagonal)	55 (100)
Left circumflex (obtuse marginal)	27 (49.1)
Right coronary artery	25 (45.5)
Right dominance	49 (89.1)
Basic echocardiographic data (biplane method)	
LVESVI (mL/m^2^)	94.7 ± 38.1
LVEDVI (mL/m^2^)	62.8 ± 33.4
LVEF (%)	35.3 ± 8.9
Basic CMR data	
LVESVI (mL/m^2^)	86.2 ± 38.0
LVEDVI (mL/m^2^)	129.1 ± 41.1
LVEF (%)	34.3 ± 8.1
Stroke volume index (mL/m^2^)	41.9 ± 8.94
Cardiac output index (L/min/m^2^)	3.0 ± 0.7

Values are presented as mean ± standard deviation or number (%)

CMR, cardiac magnetic resonance; LVEDVI, left ventricular end-diastolic volume index; LVEF, left ventricular ejection fraction; LVESVI, left ventricular end-systolic volume index

Given the ischemic nature of the myocardial status in our population (where distal segments are affected first), and that 100% of our population had a significant left anterior descending lesion, segmental viability (defined as segments with > 50% subendocardial enhancement on LGE-CMR), showed a progressive decline from the basal (75.4%) to mid (58.1%) and apical (31.6%) segments. The detailed segmental viability is presented in Table S1.

LV volumetric parameters derived from echocardiography were significantly lower than those obtained by CMR (Tables 2, 3). Correlation analysis demonstrated very strong agreement for LV end-diastolic volume (LVEDV) and LV end-systolic volume (LVESV), and strong agreement for LVEF. Bland–Altman analysis confirmed the underestimation of LV volumes by echocardiography, with a mean bias of –34.4 mL for LVEDV and –23.4 mL for LVESV, but a negligible bias for LVEF (–0.12%).

**Table 2:** Intermodality correlation between conventional left ventricular volumes and function

Parameter	r	P-value	Degree
LVEDV	0.939	< 0.001	Very strong
LVESV	0.932	< 0.001	Very strong
LVEF	0.769	< 0.001	Strong

LVEDV, left ventricular end-diastolic volume; LVEF, left ventricular ejection fraction; LVESV, left ventricular end-systolic volume

**Table 3:** Bland–Altman agreement between conventional left ventricular volumes and function

Parameter	Mean bias	95% LoA
LVEDV (mL)	–34.4	–62.1 to –6.6
LVESV (mL)	–23.4	–50.8 to 3.9
LVEF (%)	–0.12	–11.9 to 11.6

Mean bias was calculated as the difference between the echocardiography value and the cardiac magnetic resonance value (echocardiography – cardiac magnetic resonance)

LoA, limit of agreement; LVEDV, left ventricular end-diastolic volume; LVEF, left ventricular ejection fraction; LVESV, left ventricular end-systolic volume

### Intermodality correlation in GLS and segmental strain

**Fig. 4:**
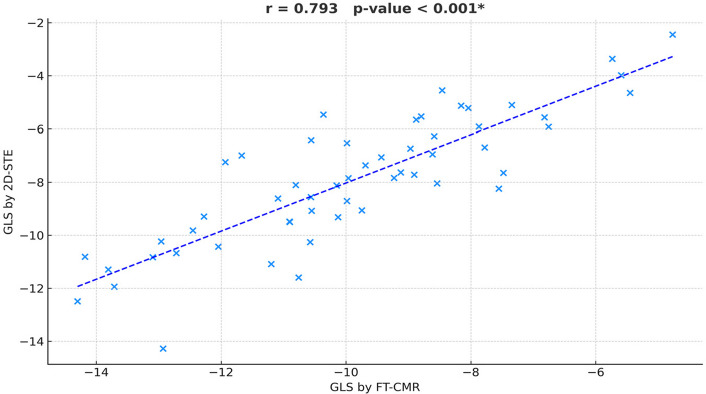
Correlation between global longitudinal strain (GLS) measured by 2D speckle-tracking echocardiography (2D-STE) and feature-tracking cardiac magnetic resonance (FT-CMR)

The GLS demonstrated a strong and statistically significant correlation between FT-CMR and 2D-STE (r = 0.793, P = 0.001) (Fig. 4). Segmental strain analysis showed more variability. Moderate correlations were observed in mid-inferoseptal, mid-inferior, mid-anterolateral, and apical segments (r = 0.382 to 0.467). Most other segments, particularly at the basal level, showed weak or poor correlation, as shown in Table 1.

### Intermodality agreement in GLS and segmental strain

Regarding the GLS, Bland–Altman analysis revealed a mean bias of + 0.98%, indicating that FT-CMR tends to yield slightly higher (less negative) GLS values compared with 2D-STE. The 95% LoAs ranged from –3.2% to + 5.1%, calculated as the mean difference ± 1.96 × SD of the differences. This corresponds to an approximate SD of 2.1%, suggesting that individual GLS measurements between the two modalities may differ by up to ± 4% to 5% in most cases (Table 4).

**Table 4:** The segmental and global longitudinal strain values measured by FT-CMR and 2D-STE, correlation coefficients, mean differences, p-values, and Bland–Altman limits of agreement

Segment	Mean FT-CMR	Mean echocardiography	r	Mean difference (bias)	P-value	95% LoA
Basal anterior	–11.38	–10.75	0.240	–0.639	0.578	–17.2 to 15.9
Basal anteroseptal	–12.16	–9.38	0.028	–2.782	0.047	–22.7 to 17.2
Basal inferoseptal	–14.1	–12.55	0.266	–1.297	0.189	–16.9 to 14.1
Basal inferior	–20.18	–14.55	0.197	–5.629	0.001	–24.9 to 13.6
Basal inferolateral	–22.15	–13.22	0.317	–8.937	< 0.001	–29.6 to 11.7
Basal anterolateral	–20.35	–11.53	0.276	–8.456	< 0.001	–25.3 to 8.0
Mid anterior	–9.26	–9.31	0.032	0.049	0.965	–16.1 to 16.1
Mid anteroseptal	–5.36	–8.96	0.068	3.601	0.008	–11.1 to 18.3
Mid inferoseptal	–5.27	–12.49	0.382	7.319	< 0.001	–4.4 to 18.8
Mid inferior	–5.36	–15.09	0.383	9.731	< 0.001	–2.3 to 21.7
Mid inferolateral	–6.87	–11.96	0.467	5.094	< 0.001	–8.0 to 18.2
Mid anterolateral	–7.23	–10.62	0.143	3.523	0.001	–11.0 to 17.9
Apical anterior	–4.79	–5.13	0.267	0.337	0.684	–11.6 to 12.3
Apical septal	–4.47	–8.31	0.214	3.838	< 0.001	–8.2 to 15.9
Apical inferior	–3.78	–7.93	0.141	4.143	< 0.001	–9.0 to 17.3
Apical lateral	–4.12	–6.73	0.080	2.609	0.002	–9.2 to 14.4
Apex	–3.76	–7.02	0.382	3.136	< 0.001	–6.7 to 13.2
LV GLS	–9.41	–10.39	0.793	0.979	0.001	–3.2 to 5.1

GLS, global longitudinal strain; LV, left ventricle

**Fig. 5:**
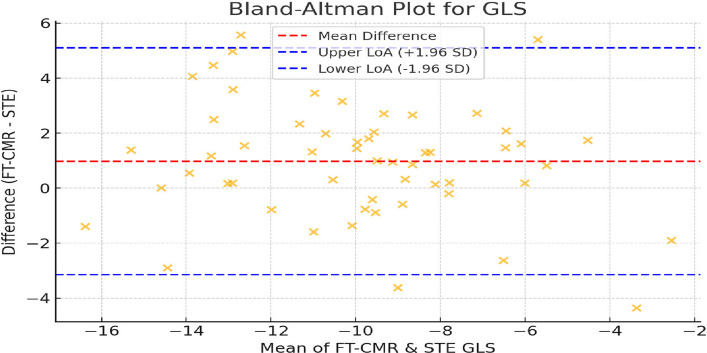
Bland–Altman plot comparing global longitudinal strain (GLS) derived from feature-tracking cardiac magnetic resonance (FT-CMR) and 2D speckle-tracking echocardiography (2D-STE). The red dashed line represents the mean difference (bias, + 0.98%), and the blue dashed lines indicate the 95% limits of agreement (–3.2% to + 5.1%). The random distribution of data points suggests the absence of proportional bias between methods

Despite the strong linear correlation between FT-CMR and 2D-STE GLS values (r = 0.793, P = 0.001), the relatively wide LoAs indicate that the two methods, while related, are not perfectly interchangeable for individual patient assessment. However, the absence of a systematic trend in the Bland–Altman plot supports good overall agreement for global function estimation across the full functional spectrum, as shown in Fig. 5.

In contrast to the overall good agreement for global strain values, segmental analysis revealed variable intermodality consistency. The basal inferolateral, basal anterolateral, and mid-inferior segments showed the greatest discrepancies, with large mean differences and wide LoAs, indicating considerable variability between modalities. In contrast, several mid-ventricular and apical regions, including the mid-inferoseptal, mid-inferolateral, and apical segments, showed better agreement, with smaller mean differences and narrower LoAs (apex, 3.136%; LoA, –6.7% to 13.2%). Conversely, certain segments, such as the mid-anterior (0.049%) and apical anterior exhibited minimal bias but broad LoAs, suggesting inconsistent measurements despite the small mean difference (Table 4). Overall, these findings highlight regional variability in intermodality agreement. When statistically exploring the potential segment-level correlations between intermodality agreement and viability, data did not show a simple, consistent statistical relationship. Generally, the potentially more viable segments, namely basal, lateral, and inferior, demonstrated the poorest concordance between modalities. However, despite regional variations, none of the segments showed sufficiently consistent agreement to be considered dependable.

Table 4 shows the GLS and segmental strain values measured by FT-CMR and 2D-STE, along with their correlation coefficients, mean differences, P-values, and Bland–Altman LoAs.

## Discussion

We assessed the relationships between GLS and segmental longitudinal strain measurements derived from 2D-STE and FT-CMR in patients with IHD and reduced ejection fraction.

In this group, accurate myocardial deformation assessment is clinically important yet technically challenging. While myocardial strain assessment using 2D-STE and FT-CMR is well established, direct intermodality comparison in this patient group remains limited, which research predominantly examining mixed heart failure cohorts or nonischemic populations. By focusing on this specific disease substrate, this study provides disease-specific insights into the feasibility, agreement, and limitations of strain measurements in the context of chronic postischemic remodeling.

We found that the two methods showed a strong positive correlation in GLS values (r = 0.793, P < 0.001), suggesting that both modalities track global systolic deformation similarly. However, segmental analysis revealed weak to moderate correlations. Regarding agreement, the LoAs across most myocardial regions were relatively wide, and more reliable on the segmental rather than the global level. This indicates that, while both techniques identify similar global deformation patterns, their absolute segmental strain values differ substantially, rendering them not interchangeable for individual patient assessment. However, they may provide comparable assessments of global myocardial function in patients with ischemic heart disease.

The absence of a proportional bias in the Bland–Altman analysis suggests that the discrepancy between techniques is consistent across different levels of myocardial dysfunction, reinforcing the reliability of both for global function estimation. Nonetheless, inherent technical differences in spatial and temporal resolution, tracking algorithms, and image quality likely contribute to the observed variability, especially at the segmental level. Overall, while both FT-CMR and 2D-STE provide valuable insights into myocardial deformation, they should be viewed as complementary rather than interchangeable tools in the functional assessment of ischemic cardiomyopathy.

When comparing intermodality agreement in LV volumes and function with that of strain, Bland–Altman analysis showed narrower LoAs for GLS than for LV volumes or LVEF, indicating superior concordance between echocardiography and CMR for GLS. This reflects the robustness of longitudinal deformation assessment, as GLS depends less on endocardial border definition and geometric assumptions than volumetric indices and is less affected by spatial resolution or image plane variations between modalities.

Previous studies have similarly shown that GLS provides a more reproducible and reliable measure of LV function than ejection fraction, independent of operator experience or imaging technique [10, 11].

The strong correlation for GLS aligns closely with the findings of Valente et al. [12], who reported a high correlation (r = 0.826) between GLS values measured by FT-CMR and 2D-STE in patients with acute ST-elevation myocardial infarction, with a mean bias of –1.09% and LoAs of ± 4.2%. Although they tested this agreement in acute IHD rather than in the chronic phase, as in our study, our data still showed comparable patterns. Zhao et al. [13] evaluated the feasibility of GLS measurement from 3D modeling of cine CMR images compared with 2D-STE in a variety of patients, including 48 healthy controls with LVEF range 53% to 74%, 30 patients with nonischemic cardiac disease with an LVEF range 25% to 77%, and two heart transplant recipients with LVEF 53% and 58%. They found a small intermodality bias (< 1%) and LoAs between –6.3% and 5.5%. These values are consistent with our study, although among different population groups.

Ananthapadmanabhan et al. [14] conducted a comparative study in 100 patients with recent myocardial infarction, reporting a GLS bias of only 0.8% between the modalities. Their study included multilayer strain analysis, and although they noted some discrepancies between endocardial, mid-myocardial, and epicardial strains, GLS remained consistent between FT-CMR and 2D-STE. Likewise, Obokata et al. [15] compared GLS measurements by FT-CMR, 2D-STE, and 3D-STE and found excellent correlation between FT-CMR and 2D-STE (r = 0.83). Their results in a heterogeneous patient population further highlight the reliability of FT-CMR in assessing global myocardial function across a wide range of clinical scenarios.

Despite the consistency in GLS, segmental strain agreement was significantly more variable in our study. Several segments, particularly the basal inferolateral and basal anterolateral segments, showed wide LoAs (e.g., basal inferolateral, –29.6% to 11.7%) and substantial mean differences. This variability was also observed in Valente et al. [12]. Compared to our results, and even though they observed numerically better correlations, the results of both studies reflect a wide range of variability in segmental strain, in contrast to global strain. They attributed this variability to differences in spatial resolution, tracking mechanics, and myocardial scar burden, which may alter local wall motion and affect tracking accuracy. Similarly, Lamacie et al. [16] found weak segmental agreement between FT-CMR and STE (r = 0.25) in patients with thalassemia major. Their study emphasized the impact of myocardial iron overload and fibrosis on segmental tracking accuracy, suggesting that regional tissue characteristics play a significant role in segmental strain variability.

Further support for this relationship comes from the work of Orwat et al. [17], who examined both healthy individuals and patients with left ventricular hypertrophy. They reported moderate agreement between FT-CMR and 2D-STE for segmental strain (r = 0.57), with discrepancies even in healthy controls. These findings imply that modality-based differences, rather than pathology alone, contribute to segmental strain variability. Taha et al. [18], who compared right ventricular longitudinal strain between FT-CMR and 2D-STE, found wide LoAs (± 11.8%) and only moderate correlation. Interestingly and in contrast to previous studies, they noted better agreement in patients with diseased right ventricles compared to healthy subjects, speculating that more pronounced myocardial deformation in damaged segments may improve tracking robustness. This may partially explain our observation that apical and mid-inferoseptal segments (often more affected in ischemic cardiomyopathy) exhibited better agreement than some basal segments.

Based on these findings, the observed discrepancies can be attributed to a range of technical and physiological issues. FT-CMR is advantaged by higher spatial resolution and sharper endocardial border definition, allowing for more accurate delineation of the myocardium. Nonetheless, it is less available, more costly, and generally suffers from lower temporal resolution than echocardiography. In contrast, 2D-STE is more widely available and has the advantage of high temporal resolution but is dependent on optimal acoustic windows. In addition, variance between strain calculation algorithms (nonrigid elastic registration for FT-CMR vs. speckle tracking for 2D-STE), hand contouring variability, and variation between frame rates can all lead to discord between segmental readings [17, 19].

Intervendor variability remains a key limitation of myocardial strain imaging. Segmental strain measurements can differ by 4% to 5% across echocardiographic platforms due to proprietary tracking algorithms, with regional measurements showing wider discrepancies than global indices. Consensus initiatives, such as the European Association of Cardiovascular Imaging–American Society of Echocardiography (EACVI-ASE) Strain Standardization Task Force, have improved GLS agreement, but residual variability persists, particularly for segmental and regional strain. Similar inter-software variability occurs in FT-CMR, emphasizing that strain values are not directly interchangeable across platforms [20].

The clinical implications of these differences are multifaceted. First, strain values obtained from different vendors or software should not be used interchangeably, especially in longitudinal follow-up or multicenter studies, without accounting for systematic biases. Second, the establishment of vendor-specific normal ranges and cutoff values may be necessary to support accurate interpretation and prognostication, as uniform thresholds may misclassify strain severity when applied across platforms [21].

Furthermore, the intrinsic heterogeneity of ischemic cardiomyopathy, characterized by variable degrees of scarring, wall thinning, and residual ischemia, represents an important determinant of intermodality variability in addition to technical factors, making intramodality variability biologically plausible. Prior studies have consistently demonstrated that regional strain values deteriorate in proportion to increasing LGE burden and transmurality, reflecting irreversible myocardial injury, altered fiber architecture, and mechanical tethering of adjacent viable myocardium [22].

Although both 2D-STE and FT-CMR demonstrate significantly lower strain values in nonviable (scarred) segments compared with viable ones, the literature provides limited and controversial data on how viability specifically influences intermodality agreement. FT-CMR strain has been shown to differentiate viable from nonviable myocardium, suggesting better sensitivity to scar, yet direct comparisons of intermodality agreement across viability categories remain scarce [12].

Notably, in accordance with the pattern noted in our study, Ananthapadmanabhan et al. [14] observed moderate overall correlation between 2D-STE and FT-CMR in post–myocardial infarction patients, with greater variability in segments retaining viability. These findings suggest that paradoxically lower agreement in viable regions may reflect subtle motion differences and tracking limitations inherent to echocardiography when compared with CMR.

Segmental differences between FT-CMR and 2D-STE raise the question of which modality more accurately reflects myocardial ischemia. In this study, direct validation against ischemia was limited by the absence of physiological lesion assessment or stress perfusion imaging. Although coronary angiography was available, anatomical stenosis severity alone is an imperfect surrogate for ischemic burden. Therefore, we deliberately avoided inferring superiority of either modality based solely on angiographic findings.

The literature indicates that myocardial strain is a sensitive marker of ischemia-induced dysfunction. STE can detect regional and global strain abnormalities associated with significant coronary disease, particularly during stress, but is limited by image quality and segmental tracking challenges. FT-CMR benefits from superior spatial resolution and integrated tissue characterization, yet lower temporal resolution and post-processing complexity may affect segmental sensitivity. Overall, FT-CMR better reflects chronic ischemic injury, whereas 2D-STE remains valuable for dynamic ischemia detection. Consequently, both modalities provide complementary information, and their differing technical characteristics likely contribute to intermodality variability, especially at the segmental level [15, 17]. These observations support cautious interpretation of regional strain and the need for modality-specific reference standards and integrated functional ischemia assessment in future studies.

This study has several limitations. IHD was defined based on anatomical coronary angiography rather than functional assessment, acknowledging that stenosis severity does not always reflect true ischemic burden. The relatively small sample size and single-center design may limit generalizability. Although imaging was performed within 1 week, minor variations in loading conditions or hemodynamics could have influenced strain measurements. CMR-derived myocardial wall thickness may also affect intermodality agreement through tracking accuracy, but quantitative data were unavailable, preventing formal analysis. Future studies with larger, multicenter cohorts, standardized post-processing, and incorporation of LGE, strain, and functional ischemia indices are needed to validate these findings and delineate more detailed mechanistic and clinical implications.

## Conclusions

In patients with IHD and reduced LVEF, GLS derived from FT-CMR and 2D-STE shows strong correlations and better intermodality agreement than conventional volumetric and functional indices. However, variability persists in segmental strain measurements, particularly in the basal regions.

The LoAs observed in our study are comparable to those reported in previous research, although in different patient cohorts, and remain relatively wide from a clinical standpoint. This suggests that while the two techniques are correlated, they cannot be considered interchangeable for individual GLS assessment, particularly when small changes in strain carry diagnostic or prognostic implications.

These results highlight the fact that the two modalities reflect similar physiological trends but differ in absolute values, likely due to intrinsic methodological differences. Clinically, this suggests that either technique may be used to evaluate global LV systolic performance, but consistent use of a single modality is recommended for longitudinal follow-up or serial viability assessment. While GLS yields a strong, reproducible index regardless of the modality, caution should be considered when interpreting regional or segmental strain data, particularly in clinical decision-making related to segmental viability or targeted therapy.

## Supplementary Information

Additional file 1: Table S1. Left ventricular segmental viability.

## Data Availability

The datasets generated and analyzed during the current study are available from the corresponding author on reasonable request, in accordance with institutional and ethical regulations.

## Abbreviations

2D-STE: 2Dimensional speckle-tracking echocardiography
AHA: American Heart Association
CMR: Cardiac magnetic resonance
FOV: Field of view
FT-CMR: Feature-tracking cardiac magnetic resonance
GLS: Global longitudinal strain
ICC: Intraclass correlation coefficients
IHD: Ischemic heart disease
LGE: Late gadolinium enhancement
LoA: Limit of agreement
LV: Left ventricular
LVEDV: Left ventricular end-diastolic volume
LVEF: Left ventricular ejection fraction
LVESV: Left ventricular end-systolic volume
SD: Standard deviation
TE: Echo time
TR: Repetition time
