# Supplementary material for: Left ventricular strain measured by feature-tracking cardiac magnetic resonance imaging and 2D speckle-tracking echocardiography in chronic ischemic heart disease: an intermodality agreement study
Source: J Cardiovasc Imaging. 2026 Apr 9;34:8. doi: 10.1186/s44348-026-00065-w (PMC13063499; doi:10.1186/s44348-026-00065-w)
Supplement: Supplementary file 1 — Additional file 1: Table S1. Left ventricular segmental viability. [file 44348_2026_65_MOESM1_ESM.docx]

**Table S1**. Left ventricular segmental viability

| Segment | Basal (330 segments) | | Segment | Mid-cavity (330 segments) | | Segment | Apical (275 segments) | |  |
| --- | --- | --- | --- | --- | --- | --- | --- | --- | --- |
|  | **Viable** | **Non-viable** |  | **Viable** | **Non-viable** |  | **Viable** | **Non-viable** |  |
| BA | 42 (76.36%) | 13 (23.64%) | **MA** | 18 (32.73%) | 37  (67.27%) | **AA** | 13 (23.64%) | 42  (76.36%) |  |
| BAS | 32 (58.18%) | 23 (41.82%) | **MAS** | 15 (27.27%) | 40  (72.73%) | **AS** | 12 (21.82%) | 43  (78.18%) |  |
| BIS | 44 (80%) | 11 (20%) | **MIS** | 28 (50.91%) | 27  (49.09%) | **AI** | 20 (36.36%) | 35  (63.64%) |  |
| BI | 45 (81.82%) | 10 (18.18%) | **MI** | 43 (78.18%) | 12  (21.82%) | **AL** | 25 (45.45%) | 30  (54.55%) |  |
| BIL | 40 (72.73%) | 15 (27.27%) | **MIL** | 42 (76.36%) | 13  (23.64%) | **AC** | 17 (30.91%) | 38  (69.09%) |  |
| BAL | 46 (83.64%) | 9 (16.36%) | **MAL** | 46 (83.64%) | 9  (16.36%) | ---- | ---- | ---- |  |
| Total | 249(75.4%) | 81(24.5%) |  | 192(58.1%) | 138(41.8%) |  | 87(31.6%) | 188(68.3%) |  |
| Total  Viable | 528 (56.47 %) | | | | | | | | |
| Total  non-viable | 407 (43.52 %) | | | | | | | | |

AA: Apical anterior, AI: Apical inferior, AL: Apical lateral, AS: Apical septal, BA: Basal anterior, BAL: Basal antero-lateral, BAS: Basal antero-septal, BI: Basal inferior, BIL: Basal infero-lateral, BIS: Basal infero-septal, MA: Mid anterior, MAL: Mid antero-lateral, MAS: Mid antero-septal, MI: Mid inferior, MIL: Mid infero-lateral, MIS: Mid infero-septal.
